# Identification of novel serum proteins that distinguish idiopathic recurrent aphthous stomatitis from Behcet’s disease

**DOI:** 10.7717/peerj.21511

**Published:** 2026-07-15

**Authors:** Mengya Zhu, Xinliang Mao, Yong Peng, Xianqian Huang, Baoqing Geng, Minzhi Gan, Ying Ying, Keyue Zhang, Yong Chen

**Affiliations:** 1 Department of Rheumatology and Immunology, Ningbo No. 2 Hospital, Wenzhou Medical University, Ningbo, Zhejiang, China; 2 Emergency Department, Ningbo No. 2 Hospital, Wenzhou Medical University, Ningbo, Zhejiang, China

**Keywords:** Recurrent aphthous stomatitis, Behcet’s disease, Differentially expressed protein, Proteomic analysis, Diagnosis

## Abstract

**Background:**

Recurrent aphthous stomatitis (RAS) is a chronic autoinflammatory condition marked by recurring, painful sores in the mouth. It is often confused with Behcet’s disease (BD), a rare systemic vasculitis that also presents with oral ulcers. Despite overlapping symptoms, BD has broader systemic implications, making an accurate diagnosis critical. This study aims to identify unique serum proteins that could reliably distinguish idiopathic RAS from BD.

**Methods:**

We reanalyzed our previous mass spectrometry dataset comprising blood samples from 12 BD patients, 12 individuals with idiopathic RAS, and 21 healthy controls. Differentially expressed proteins (DEPs) related to RAS were identified and examined through Kyoto Encyclopedia of Genes and Genomes and Gene Ontology pathway enrichment. A protein-protein interaction (PPI) network was created to explore functional connections among the DEPs. Validation of three RAS-related proteins was carried out using enzyme-linked immunosorbent assay (ELISA) in a separate cohort of 26 RAS patients, 26 BD patients, and 30 healthy individuals. Their diagnostic utility was then evaluated *via* receiver operating characteristic (ROC) curve analysis.

**Results:**

A total of 99 proteins showed differential expression in RAS samples but not in BD cases when compared to healthy controls—85 were upregulated, and 14 were downregulated. Enrichment analyses indicated these proteins are primarily involved in metabolic and infection-related pathways, particularly influencing keratinocyte differentiation and oxidative stress responses. PPI network analysis highlighted key metabolic and keratinocyte-related proteins as central hubs, suggesting a role in RAS pathology. ELISA validation confirmed significantly elevated levels of ANXA2, ENO1, and S100A7 in RAS patients compared to both BD patients and healthy subjects.

**Conclusion:**

Our findings identify a set of RAS-related serum proteins with potential diagnostic value. These serum proteins may enhance the clinical differentiation of RAS from BD, aiding in more accurate and timely patient care.

## Introduction

Recurrent aphthous stomatitis (RAS), commonly known as canker sores, is a chronic condition marked by recurring, painful ulcers in the oral cavity. These lesions typically develop on the labial and buccal mucosa, gingiva, tongue, and palate, appearing as round or oval breaks in the oral epithelium (Scully & Porter, 2008). Although the precise cause of RAS remains elusive, various factors such as mucosal trauma, genetic susceptibility, immune dysregulation, nutritional deficiencies, and psychological stress are believed to contribute to its onset (Plewa & Chatterjee, 2024). RAS affects individuals across all age groups (Manfredini et al., 2021; Queiroz et al., 2018; Rivera, 2019). While it is noncontagious and often resolves on its own, the associated pain and discomfort can significantly impact quality of life. Current treatment strategies, ranging from topical agents to oral rinses and analgesics, are primarily aimed at symptom management (Belenguer-Guallar, Jimenez-Soriano & Claramunt-Lozano, 2014; Manfredini et al., 2021).

Behcet’s disease (BD) is a rare, systemic autoimmune disorder characterized by vasculitis that can affect blood vessels of all sizes and types. It involves multiple organ systems, including the mucous membranes, skin, joints, eyes, central nervous system, and gastrointestinal tract (Adil, Goyal & Quint, 2024; Sakane et al., 1999). Though its exact cause remains unknown, genetic predisposition, epigenetic mechanisms, and environmental triggers collectively initiate abnormal immune responses in susceptible individuals (Mattioli et al., 2021). BD is more prevalent in regions along the ancient Silk Road, spanning from East Asia to the Mediterranean, suggesting a geographical pattern to its distribution (Leonardo & McNeil, 2015). Similar to RAS, there is no definitive cure for BD; treatment is focused on reducing inflammation and controlling symptoms (Alpsoy et al., 2021).

Oral ulcers resembling RAS are a common feature in BD patients. However, similar lesions can also appear in the context of gastrointestinal disorders such as Crohn’s disease and ulcerative colitis (Cui, Bruce & Rogers 3rd, 2016). This overlap complicates the diagnostic process, highlighting the need for specific biomarkers that can distinguish BD-related ulcers from idiopathic RAS.

To date, proteomic investigations of RAS have been conducted almost exclusively using salivary samples, implicating pathways related to vitamin metabolism, bacterial response, and immunogenic cell death in RAS pathogenesis (Cofré-Leiva et al., 2023; Franco et al., 2025; Hernández-Olivos et al., 2021). While these salivary-based studies provide valuable insights into local oral pathophysiology, they also highlight a notable gap: the role of circulating serum proteins in RAS remains largely unexplored. Given that serum reflects systemic alterations, serum-based proteomic profiling could offer a complementary perspective and uncover additional biomarkers with diagnostic or prognostic value.

To fill these gaps, we employed proteomic analysis using patient serum samples. Notably, the mass spectrometry dataset used here has been previously reported, where our primary focus was on identifying serum proteins associated with BD and its systemic inflammatory characteristics (Zhu et al., 2025). While our previous work identified a few serum proteins unique to BD, limited information exists regarding proteins related to idiopathic RAS. To address this, we reanalyzed our existing dataset using a different analytical strategy, aiming to identify differentially expressed proteins (DEPs) found in RAS but not in BD serum samples. This approach enabled us to uncover molecular features specific to RAS, representing a novel perspective beyond the original analysis. Subsequent bioinformatic analysis revealed that these RAS-related DEPs are primarily involved in metabolic regulation, inflammatory responses, and keratinocyte differentiation. To validate these findings, we used enzyme-linked immunosorbent assay (ELISA) to measure the levels of three hub proteins identified through protein-protein interaction (PPI) analysis, and confirmed their potential diagnostic value.

## Methods

### Human subjects

The cohort used for proteomic analysis was previously described in detail (Zhu et al., 2025). More detailed clinical information of BD patients is shown in Table 1, Tables S1 and S2. These patients were first hospitalized for BD symptoms during the active stage of the disease and had not received any prior treatment. Similarly, the RAS patients had not received any treatment, and their age and sex information are shown in Tables S3 and S4. Diagnosis of BD was established according to the International Criteria for Behcet’s Disease (ICBD) (International Team for the Revision of the International Criteria for Behcet’s Disease, 2014). The Behcet’s Disease Current Activity Form (BDCAF) score was used to assess disease activity, with patients scoring ≥2 considered to have active BD (Bhakta et al., 1999; Lawton et al., 2004). Diagnosis of RAS is primarily based on clinical evaluation, as no specific laboratory tests are currently available. It is characterized by recurrent, self-limiting oral ulcers presenting with a central depression, a yellow pseudomembrane, surrounding erythema, and associated pain. A definitive diagnosis requires excluding other conditions that may cause similar oral ulcerations (Scully, Gorsky & Lozada-Nur, 2003; Tarakji et al., 2015). Individuals with RAS included in the study had no clinical evidence of BD, malignancies, or other rheumatic or infectious diseases and were not biologically related. For ELISA validation, peripheral blood serum samples were collected from 26 patients with idiopathic RAS and 26 BD patients, all of whom were diagnosed and treated at our institution between February and October 2024. An additional group of 30 healthy volunteers served as controls. The age and sex distributions of participants in the proteomic analysis and ELISA cohorts are presented in Tables S5 and S6. Comparative analysis revealed no significant differences among the three groups (*P* > 0.05), confirming the comparability of the study populations. The study protocol was approved by the Ethics Committee of Ningbo No. 2 Hospital (Approval No. YJ-KYSB-NBEY-2020-144-01). Informed written consent was obtained from all participants in accordance with the ethical principles of the Declaration of Helsinki (1975, revised in 2013).

**Table 1:** Summary of the clinical characteristics of BD patients.

Clinical characteristic	Proteomics (*N* = 12)	ELISA (*N* = 26)
BDCAF score, median [IQR]	4 [3, 5]	4 [3, 4]
BDCAF component, *N* (%)	
Headache	3 (25.0)	6 (23.1)
Oral ulceration	11 (91.7)	24 (92.3)
Genital ulcer	5 (41.7)	14 (53.8)
Erythema	7 (58.3)	17 (65.4)
Skin pustule	3 (25.0)	3 (11.5)
Joint involvement	
Joints-Arthralgia	5 (41.7)	14 (53.8)
Joints-Arthritis	2 (16.7)	3 (11.5)
Gastrointestinal symptoms	
Nausea/vomiting/abdominal pain	3 (25.0)	5 (19.2)
Diarrhea + altered/frank blood per rectum	1 (8.3)	1 (3.8)
New active eye symptom	4 (33.3)	7 (26.9)
New active nervous system involvement	2 (16.7)	3 (11.5)
New active major vessel inflammation	2 (16.7)	1 (3.8)

### Bioinformatic analysis

During proteomic analysis, every four consecutively numbered patient samples (*e.g.*, BD-1 to BD-4 pooled as replicate 1) were combined in equal volumes to generate one biological replicate for the BD and RAS groups. For the control group, every seven consecutively numbered samples were pooled to form three biological replicates. Differentially expressed proteins (DEPs) between two indicated datasets were identified as previously reported (Zhu et al., 2025). Proteins with a fold change (FC) ≤ 0.83 were considered downregulated, whereas those with an FC ≥ 1.2 were considered upregulated (Serang et al., 2013). Normality was tested using the Shapiro–Wilk test. Unpaired bilateral Student’s *t*-tests were initially applied to evaluate statistical significance. DEPs (*P*-value < 0.05) were further validated by the Benjamini–Hochberg method using R (v4.2.1), and an FDR < 0.05 is used as the threshold of statistical significance (File S1). Functional enrichment of DEPs was conducted using the R package ‘clusterProfiler’ (v4.0) (Wu et al., 2021) within R for Gene Ontology (GO) and Kyoto Encyclopedia of Genes and Genomes (KEGG) pathway analysis. Venn diagrams were created using the ‘VennDiagram’ package (v1.7.3). PPI networks were constructed *via* the STRING database (https://cn.string-db.org/). Key hub proteins within these networks were identified using Cytoscape software (v3.10.2) and its cytoHubba plugin (v0.1) (Chin et al., 2014; Shannon et al., 2003). Ranking of hub proteins was performed using the “Degree” algorithm from cytoHubba, which evaluates nodes based on the number of direct interactions.

### ELISA

To validate the differential expression of ANXA2, ENO1, and S100A7 across RAS, BD, and healthy controls, ELISA was performed using commercial kits from Jiangsu Kete Biotech (Jiangsu, China), following the manufacturer’s protocols. Their catalog numbers are as follows: KT9930-A (ANXA2), KT9708-A (ENO1), and KT0715-HA (S100A7). Absorbance values (OD_450_) were measured using a SpectraMax M5 microplate reader (Molecular Devices, San Jose, CA, USA). Data analysis was conducted using R, with visualization *via* the ‘ggplot2’ package (v3.5.1). Each data point represents the average of three technical replicates. Normality was tested using the Shapiro–Wilk test. Statistical comparisons among groups were conducted using one-way ANOVA with *post hoc* Tukey’s HSD test. *P*-values < 0.05 were considered statistically significant.

### Receiver operating characteristic analysis

The potential diagnostic performance of ELISA-validated RAS-related serum proteins was evaluated through receiver operating characteristic (ROC) curve analysis using Python (v3.14).

## Results

### Identification and functional analysis of RAS-related serum DEPs

**Figure 1:**
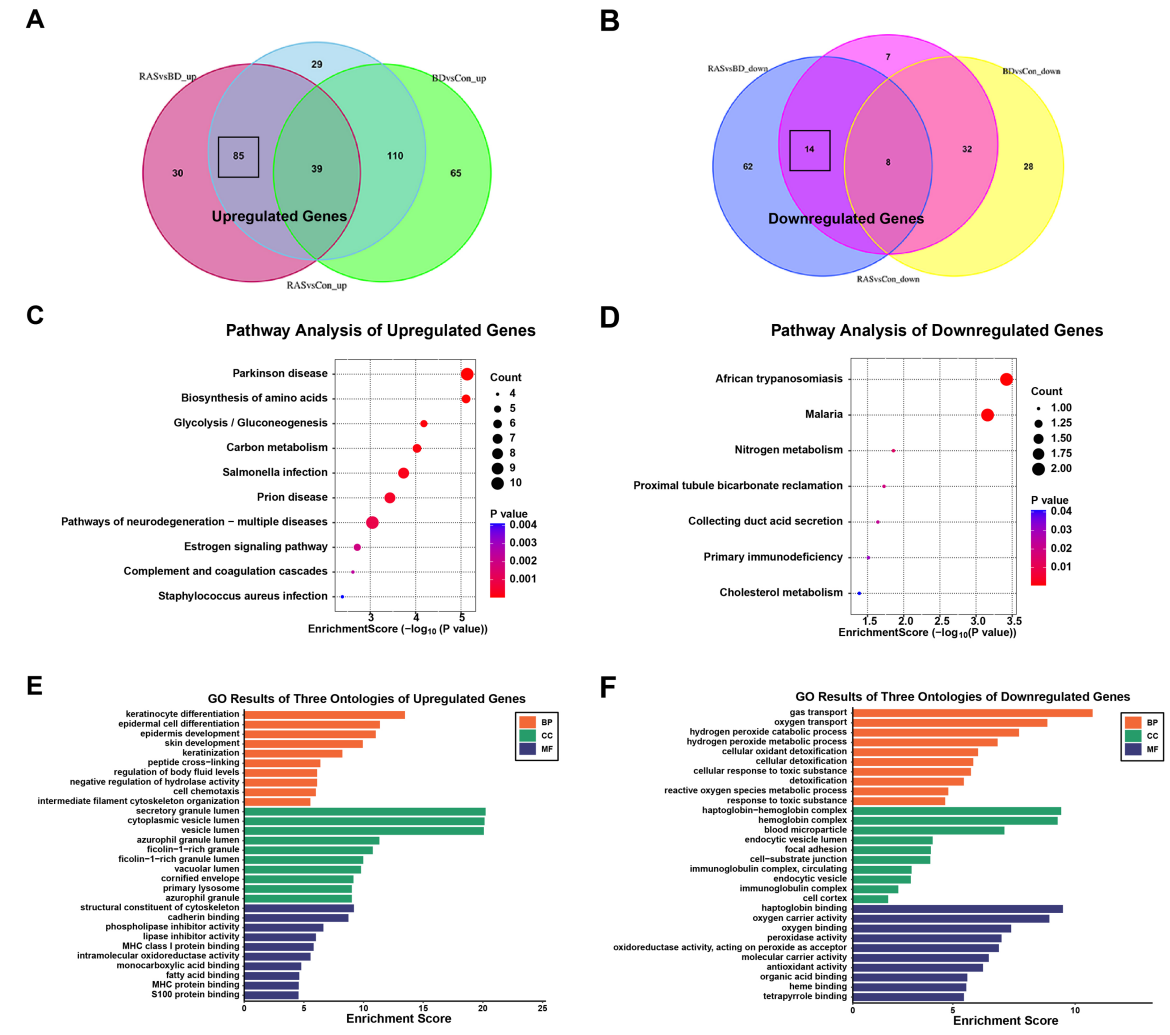
Identification and functional enrichment of RAS-specific serum DEPs. (A) Venn diagrams showing DEPs unique to RAS patients but not BD patients, compared with healthy controls (outlined by black squares). (B, C) KEGG pathway enrichment of upregulated (B) and downregulated (C) RAS-specific DEPs. (D, E) Cnetplots depicting the upregulated (D) and downregulated DEPs (E) with their associated KEGG pathways. (F and G) Barplots indicating the GO analysis results of upregulated (F) and downregulated DEPs (G) unique to RAS serum.

To isolate serum proteins uniquely associated with RAS, we focused on DEPs present in RAS patients but not in BD patients relative to healthy controls. This comparative analysis revealed 99 RAS-related DEPs, comprising 85 upregulated and 14 downregulated proteins (Figs. 1A and 1B, Table S7). KEGG pathway enrichment analysis indicated that the upregulated DEPs were predominantly involved in pathways related to Parkinson’s disease, amino acid biosynthesis, and carbon metabolism. In contrast, the downregulated proteins were linked to pathways including African trypanosomiasis, malaria, and nitrogen/cholesterol metabolism (Figs. 1C and 1D; Figs. S1A and S1B). GO analysis further categorized these DEPs. The upregulated proteins were primarily localized to the secretory granule lumen, epidermal cornified envelope, and primary lysosome (cellular component, CC) and were involved in biological processes (BPs) such as keratinocyte differentiation and cytoskeleton organization. Examples of their molecular functions (MFs) included cadherin binding and MHC protein binding (Fig. 1E).

Conversely, downregulated DEPs were mainly associated with the hemoglobin complex, blood microparticles, and focal adhesion, modulating BPs like gas transport and cellular detoxification, with MFs such as haptoglobin binding, peroxidase activity, and antioxidant function (Fig. 1F). These findings suggest that the RAS serum proteome is enriched for proteins regulating keratinocyte differentiation and depleted of proteins related to oxidative stress response, distinguishing it from BD serum.

### PPI network analysis of RAS-related DEPs

**Figure 2:**
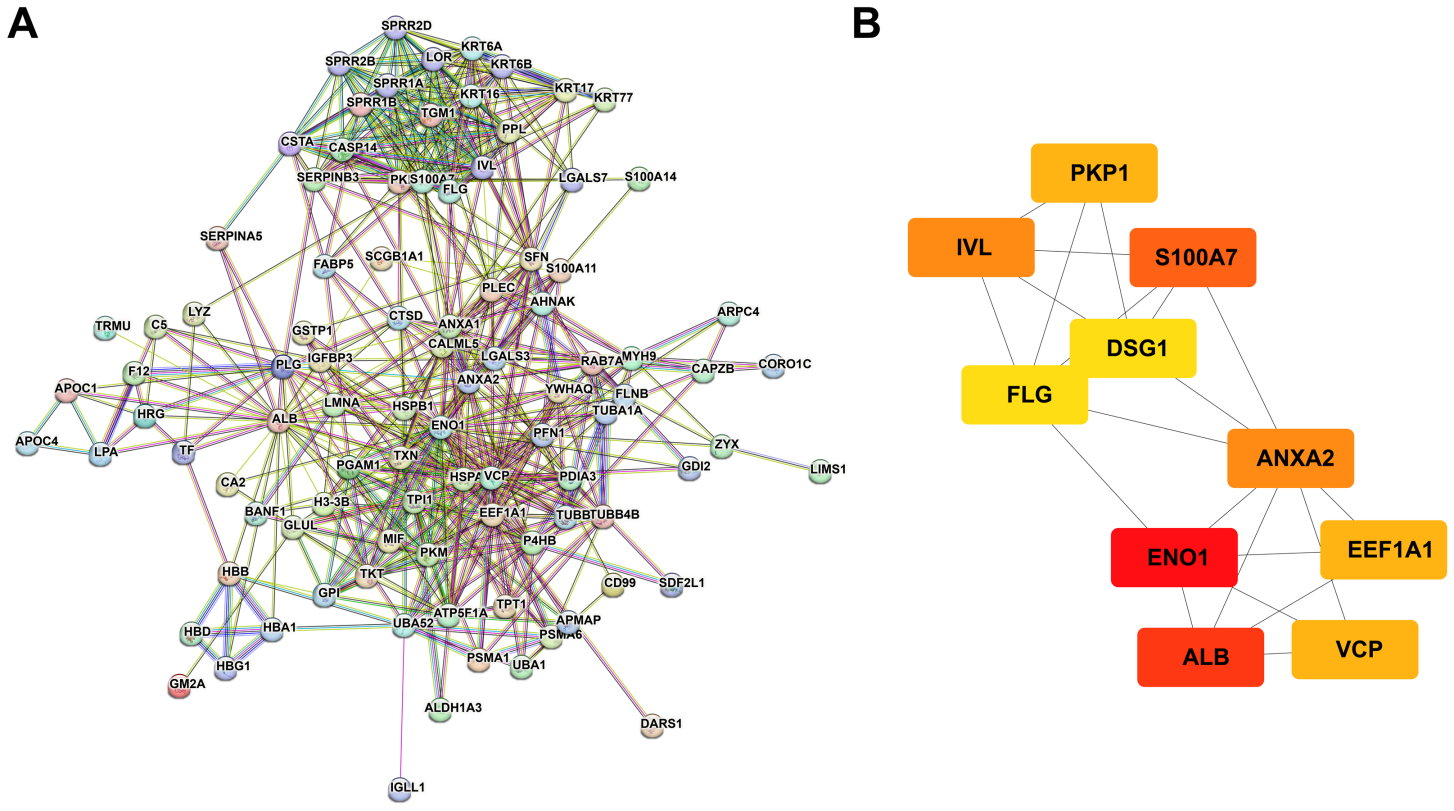
PPI network of RAS-specific DEPs. (A) PPI network constructed from RAS-specific DEPs using STRING and visualized with Cytoscape. (B) Top 10 hub proteins identified using the cytoHubba plugin, grouped into clusters associated with keratinocyte differentiation and metabolic regulation. The detailed rank information is shown in File S2.

To examine the interplay among RAS-related serum proteins, we constructed a PPI network using the STRING database, followed by visualization and hub analysis *via* Cytoscape with the cytoHubba plugin (Fig. 2A). The analysis identified two distinct clusters among the top 10 hub proteins. The first cluster, comprising PKP1, IVL, S100A7, DSG, and FLG, was associated with epidermal and keratinocyte differentiation. The second cluster, containing ANXA2, ENO1, ALB, EEF1A1, and VCP, was primarily composed of proteins involved in glycolysis and cellular metabolism (Fig. 2B). These network findings are in agreement with the enrichment analysis results and highlight potential diagnostic relevance for proteins related to keratinocyte biology and metabolic regulation in RAS.

### Validation of key RAS-related serum DEPs by ELISA

**Figure 3:**
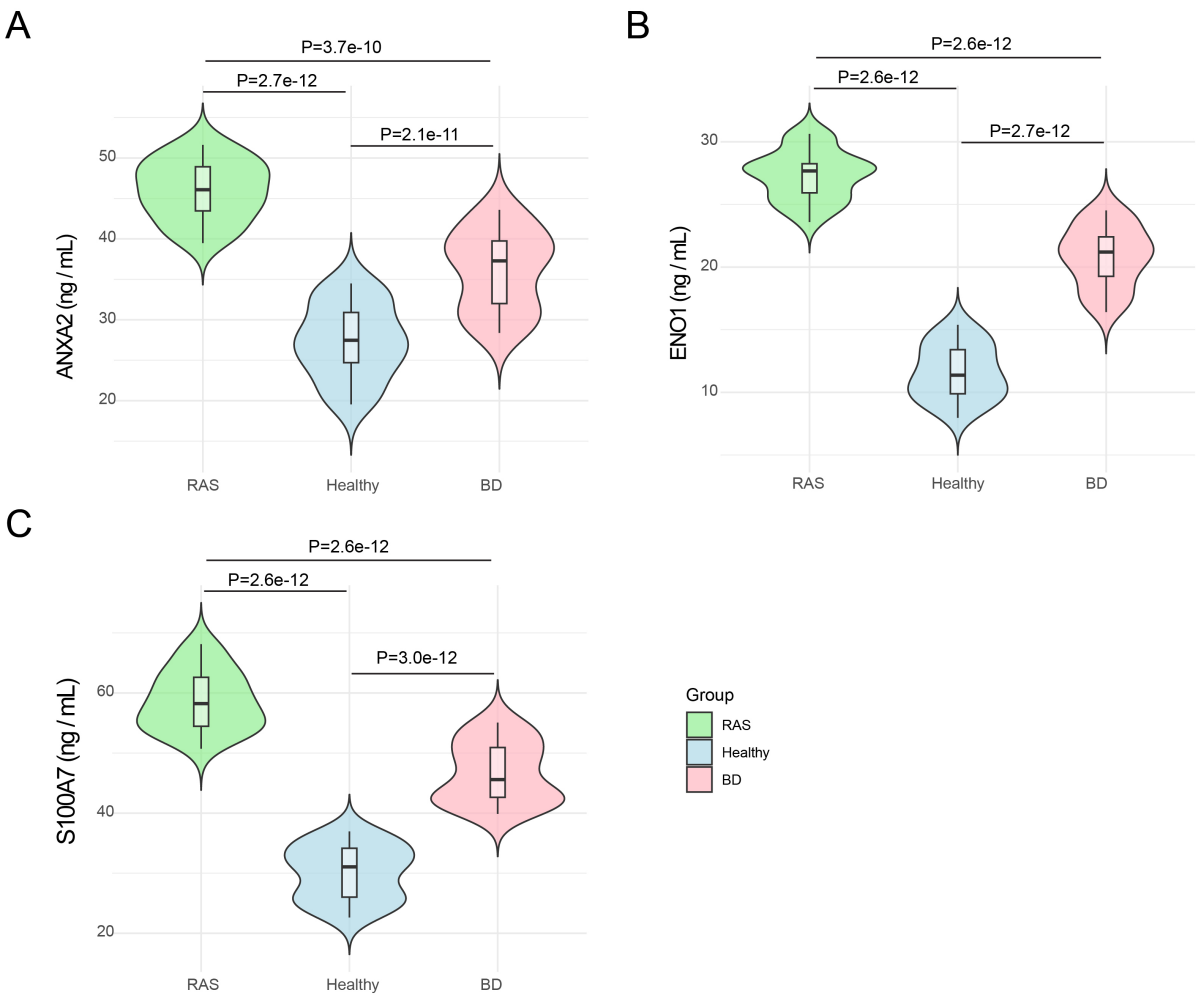
Validation of RAS-specific proteins by ELISA. (A–C) Violin plots showing serum levels of ANXA2 (A), ENO1 (B), and S100A7 (C) measured by ELISA in RAS, BD, and healthy controls. Statistical significance was determined by one-way ANOVA with *post hoc* Tukey’s HSD test.

To confirm the differential expression of key hub proteins identified in the proteomic analysis, we selected three candidates with commercially available ELISA kits (ENO1, ANXA2, and S100A7) for validation using ELISA in an independent cohort of RAS, BD, and healthy subjects. ELISA results demonstrated that all three proteins were significantly elevated in RAS serum samples compared to both BD patients and healthy controls (Figs. 3A–3C, Table S8). Interestingly, levels in BD patients were also moderately elevated compared to healthy individuals, though significantly lower than those observed in RAS.

**Figure 4:**
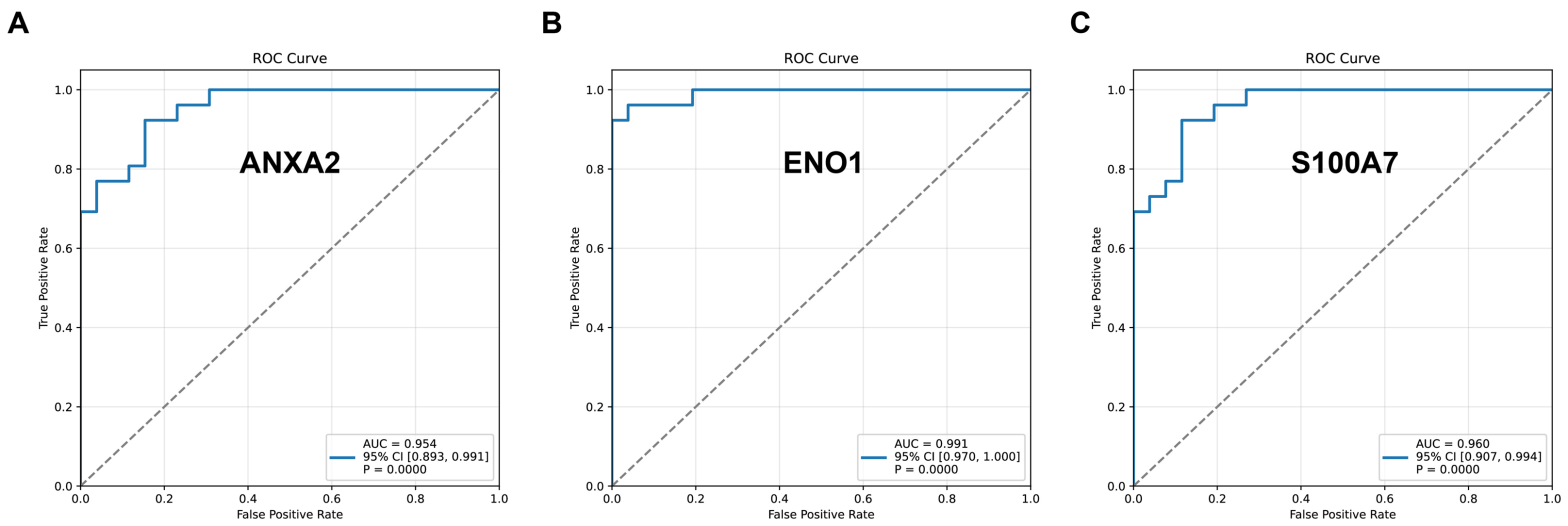
Diagnostic performance of RAS-specific serum proteins. (A–C) ROC curves for ANXA2 (A), ENO1 (B), and S100A7 (C) showing their ability to distinguish RAS from BD and healthy controls. CI, confidence interval.

To assess the diagnostic potential of these proteins, we conducted ROC curve analyses. All three markers exhibited strong diagnostic performance in distinguishing RAS from BD, with 95% confidence intervals (CIs) for the area under the curve (AUC) of 0.893–0.991 for ANXA2, 0.97–1 for ENO1, and 0.907–0.994 for S100A7, respectively (Figs. 4A–4C). These findings support the use of ENO1, ANXA2, and S100A7 as potential serum proteins for the diagnosis of RAS and its differentiation from BD.

## Discussion

RAS and BD share a key clinical hallmark, oral ulceration, making the differential diagnosis between these two autoinflammatory conditions particularly challenging, especially in the early stages. Although BD presents with multisystem involvement and systemic inflammation, its initial symptoms often mimic idiopathic RAS, leading to potential diagnostic delays and misclassification. Thus, identifying disease-specific biomarkers remains critical for accurate and timely diagnosis.

In this study, we focused on distinguishing idiopathic RAS from BD at the serum protein level by leveraging a comparative proteomic approach. By specifically screening for DEPs present in RAS but not in BD, we identified a set of 99 RAS-related serum proteins. These DEPs, when subjected to functional enrichment analysis, revealed a strong association with keratinocyte differentiation, cytoskeletal organization, and metabolic regulation.

Our functional enrichment analyses provided key insights into the underlying pathophysiology of RAS. The enrichment of proteins involved in the secretory granule lumen, epidermal cornified envelope, and lysosomal activity suggests enhanced epithelial turnover and barrier remodeling in RAS patients. Moreover, the upregulation of pathways linked to metabolic and oxidative stress-related processes highlights the possibility that oral epithelial stress responses may play a more central role in RAS pathogenesis than previously understood. Supporting this theory, proteomic analysis of RAS saliva reveals that abnormal keratinocyte apoptosis and endoplasmic reticulum stress contribute to the etiopathogenesis of RAS (Cofré-Leiva et al., 2023). However, the mechanisms by which these keratinocyte-derived components enter the circulatory system remain to be further investigated.

Our PPI network analysis further reinforced the relevance of epithelial and metabolic proteins in RAS. We identified two major clusters of hub proteins: one linked to keratinocyte and epidermal differentiation (PKP1, IVL, S100A7, DSG, FLG), and the other to glycolytic and metabolic pathways (ANXA2, ENO1, ALB, EEF1A1, VCP). These findings suggest that while immune dysregulation may contribute to RAS (Chiang et al., 2019), metabolic imbalance, alongside epithelial homeostasis as previously discussed, appears to be another key feature of the disease’s serum molecular signature. Indeed, patients with metabolic syndrome show a higher prevalence of RAS (Abdollahian, Pourzare Mehrbani & Motahari, 2020), and proteomic analysis of RAS patient saliva reveals significant metabolic dysregulation (Dong et al., 2024).

To validate these findings, we assessed the expression of three hub proteins—ENO1, ANXA2, and S100A7—*via* ELISA. Validation of the other hub proteins was not performed due to the limited availability of commercially accessible ELISA kits. Consistent with our proteomic data, these proteins were significantly elevated in RAS patient serum compared to both BD patients and healthy controls. While some elevation was also observed in BD patients, the levels remained substantially lower, supporting the notion that these proteins are more specifically associated with idiopathic RAS. ROC analysis further confirmed the high diagnostic potential of these markers, suggesting their possible utility in clinical differentiation between RAS and BD. S100A7 is an oncogene associated with oral squamous cell carcinoma (OSCC) and has been identified as a potential diagnostic biomarker for oral malignancies (Dey et al., 2016; Sood et al., 2022). ENO1 also plays an oncogenic role in OSCC, potentially by enhancing the metastatic potential through the regulation of macrophage IL-6 secretion (Lin et al., 2023; Liu et al., 2020). Furthermore, elevated serum levels of ANXA2 have been observed in OSCC patients and are negatively correlated with clinical outcomes (Zhang et al., 2017). The upregulation of these proteins in RAS serum suggests a shared involvement in oral diseases, though their exact roles in RAS pathogenesis remain to be confirmed through functional assays.

It is noteworthy that our study has several limitations. First, the sample size, though balanced across groups, was relatively small and drawn from a single center, which may limit the generalizability of the findings. Future studies with larger, multi-center cohorts are needed to validate these results. Second, while the proteomic and ELISA analyses provided consistent results, only three candidate proteins were validated, and additional RAS-related DEPs may have been overlooked. Future investigations using expanded validation panels or targeted proteomics approaches are warranted. Third, potential confounding factors, such as metabolic status and smoking, were not fully controlled. Given the involvement of metabolic pathways, subsequent studies should include well-matched cohorts and detailed clinical data to enable multivariate analysis. Fourth, while ELISA provides sensitive and quantitative measurement, it lacks information on protein isoforms and molecular weight. Complementary methods like Western blotting-based validation would strengthen the findings. Fifth, sample pooling in the proteomics discovery phase was required to ensure adequate protein detection but limited the assessment of biological variability and statistical inference; therefore, these results should be interpreted as exploratory and require validation in individual samples using additional methods such as ELISA. Finally, although we identified proteins associated with keratinocyte differentiation and metabolism, no functional experiments were performed. Further mechanistic studies are required to establish causal relationships in RAS pathogenesis.

## Conclusions

In summary, our study identifies multiple RAS-related serum proteins, distinguishing idiopathic RAS from BD. These markers, linked to keratinocyte differentiation and metabolism, provide valuable insights into the molecular features of RAS. While they may have potential as candidate biomarkers, further validation in larger cohorts is required to determine their clinical utility.

##  Supplemental Information

10.7717/peerj.21511/supp-1 Fig. S1 DEPs associated with their associated KEGG pathways (A, B) Cnetplots depicting the upregulated (A) and downregulated DEPs (B) with their associated KEGG pathways

10.7717/peerj.21511/supp-2 File S1 List of DEPs among the three conditions

10.7717/peerj.21511/supp-3 File S2 Ranking information of the top 10 hub proteins

10.7717/peerj.21511/supp-4 Table S1 Sex, age, and BDCAF score of BD patients included in proteomic analysis

10.7717/peerj.21511/supp-5 Table S2 Sex, age, and BDCAF score of BD patients included in ELISA

10.7717/peerj.21511/supp-6 Table S3 Sex and age of RAS patients and healthy volunteers included in proteomic analysis

10.7717/peerj.21511/supp-7 Table S4 Sex and age of RAS patients and healthy volunteers included in ELISA

10.7717/peerj.21511/supp-8 Table S5 Comparison of demographic characteristics of the proteomic analysis cohort

10.7717/peerj.21511/supp-9 Table S6 Comparison of demographic characteristics of the ELISA cohort

10.7717/peerj.21511/supp-10 Table S7 List of DEPs unique to RAS, rather than BD, compared to healthy serum

10.7717/peerj.21511/supp-11 Table S8 Power calculation results of the ELISA data

10.7717/peerj.21511/supp-12 Supplemental Information 12 STROBE checklist

## Abbreviations

RAS: Recurrent aphthous stomatitis
BD: Behcet’s disease
DEP: Differentially expressed protein
ELISA: Enzyme-linked immunosorbent assay
PPI: Protein-protein interaction
ICBD: International Criteria for Behcet’s Disease
GO: Gene Ontology
KEGG: Kyoto Encyclopedia of Genes and Genomes
CC: Cellular component
BP: Biological process
MF: Molecular function
OSCC: Oral squamous cell carcinoma

## Additional Information and Declarations

### Data Availability

The following information was supplied regarding data availability: The data and code are available in the Supplemental Files. The data is available at: https://www.iprox.cn//page/project.html?id=IPX0016314000. No logins or program installations are required when using the HTTP portal to download the data. We are not able to access the Zenodo platform, and the current link guarantees free access and download. The data owner and institution remain unchanged. The codes we used are directly from the official tutorial: - VennDiagram: https://cran.r-project.org/web/packages/VennDiagram/refman/VennDiagram.html. - ggplot2: https://ggplot2.tidyverse.org/articles/ggplot2.html. - clusterProlifer: https://guangchuangyu.github.io/software/clusterProfiler/documentation/.
