# Supplementary figures and images for: Identification of novel serum proteins that distinguish idiopathic recurrent aphthous stomatitis from Behcet’s disease
Source: PeerJ. 2026 Jul 15;14:e21511. doi: 10.7717/peerj.21511 (PMC13380236; doi:10.7717/peerj.21511)

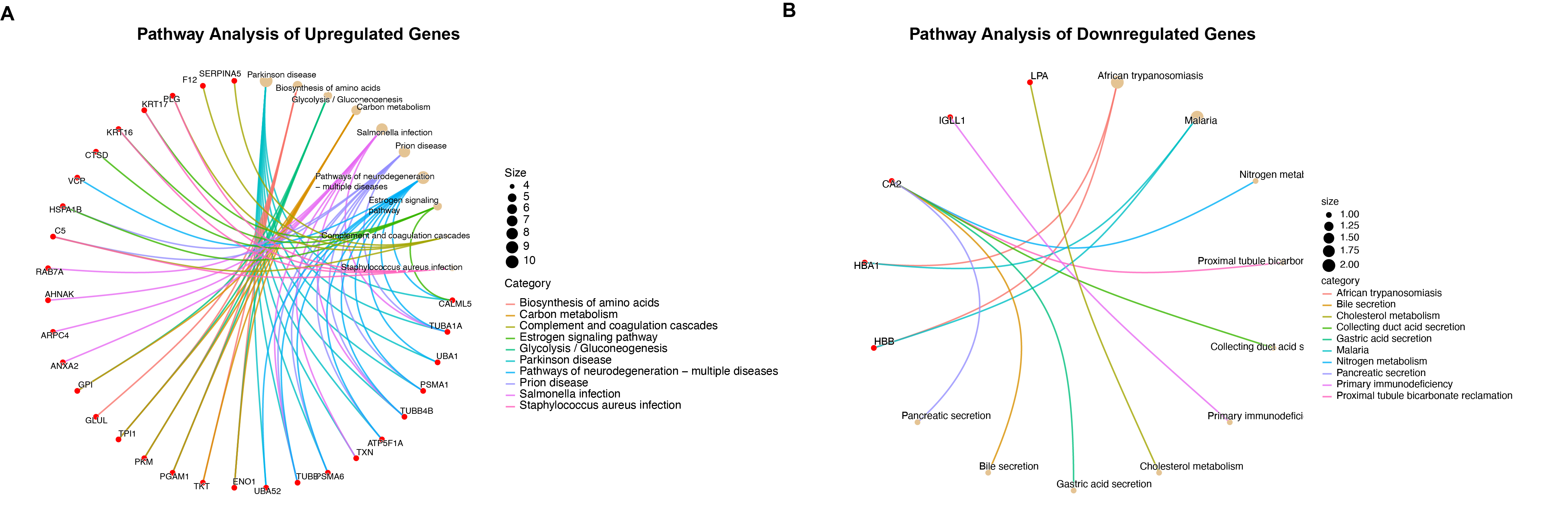

Supplement: Fig. S1 — (A, B) Cnetplots depicting the upregulated (A) and downregulated DEPs (B) with their associated KEGG pathways [file peerj-14-21511-s001.jpg]
