# Supplementary material for: Identification of novel serum proteins that distinguish idiopathic recurrent aphthous stomatitis from Behcet’s disease
Source: PeerJ. 2026 Jul 15;14:e21511. doi: 10.7717/peerj.21511 (PMC13380236; doi:10.7717/peerj.21511)
Supplement: Table S1 [file peerj-14-21511-s004.docx]

| Sex | Age | Proteomic analysis (12 cases) | BDCAF score (12 points total) | Replicate |
| --- | --- | --- | --- | --- |
| M | 26 | BD-1 | 4 | #1 |
| F | 33 | BD-2 | 5 |  |
| F | 23 | BD-3 | 3 |  |
| F | 48 | BD-4 | 2 |  |
| F | 44 | BD-5 | 4 | #2 |
| M | 38 | BD-6 | 5 |  |
| M | 66 | BD-7 | 5 |  |
| F | 62 | BD-8 | 3 |  |
| F | 54 | BD-9 | 3 | #3 |
| F | 37 | BD-10 | 5 |  |
| M | 62 | BD-11 | 3 |  |
| F | 35 | BD-12 | 4 |  |

Table S1. Sex, age, and BDCAF score of BD patients included in proteomic analysis.
