# Supplementary material for: Identification of novel serum proteins that distinguish idiopathic recurrent aphthous stomatitis from Behcet’s disease
Source: PeerJ. 2026 Jul 15;14:e21511. doi: 10.7717/peerj.21511 (PMC13380236; doi:10.7717/peerj.21511)
Supplement: Table S2 [file peerj-14-21511-s005.docx]

| Sex | Age | ELISA (26 cases) | BDCAF score (12 points total) |
| --- | --- | --- | --- |
| M | 39 | BD-1 | 4 |
| F | 26 | BD-2 | 3 |
| F | 57 | BD-3 | 4 |
| F | 54 | BD-4 | 5 |
| M | 62 | BD-5 | 3 |
| M | 45 | BD-6 | 5 |
| F | 43 | BD-7 | 3 |
| M | 51 | BD-8 | 4 |
| F | 28 | BD-9 | 5 |
| F | 37 | BD-10 | 3 |
| M | 42 | BD-11 | 3 |
| M | 67 | BD-12 | 3 |
| F | 48 | BD-13 | 4 |
| M | 32 | BD-14 | 4 |
| F | 52 | BD-15 | 3 |
| F | 59 | BD-16 | 3 |
| F | 64 | BD-17 | 7 |
| M | 43 | BD-18 | 3 |
| M | 55 | BD-19 | 5 |
| F | 16 | BD-20 | 4 |
| F | 29 | BD-21 | 4 |
| M | 40 | BD-22 | 4 |
| F | 46 | BD-23 | 2 |
| F | 34 | BD-24 | 2 |
| F | 55 | BD-25 | 3 |
| F | 59 | BD-26 | 4 |

Table S2. Sex, age, and BDCAF score of BD patients included in ELISA.
