# Supplementary material for: Identification of novel serum proteins that distinguish idiopathic recurrent aphthous stomatitis from Behcet’s disease
Source: PeerJ. 2026 Jul 15;14:e21511. doi: 10.7717/peerj.21511 (PMC13380236; doi:10.7717/peerj.21511)
Supplement: Table S3 [file peerj-14-21511-s006.docx]

| Sex | Age | IDs | Replicate |
| --- | --- | --- | --- |
| F | 32 | RAS-1 | #1 |
| F | 65 | RAS-2 |  |
| M | 49 | RAS-3 |  |
| F | 33 | RAS-4 |  |
| F | 34 | RAS-5 | #2 |
| F | 49 | RAS-6 |  |
| M | 48 | RAS-7 |  |
| M | 52 | RAS-8 |  |
| F | 25 | RAS-9 | #3 |
| F | 32 | RAS-10 |  |
| M | 56 | RAS-11 |  |
| F | 51 | RAS-12 |  |
| M | 44 | Control-1 | #1 |
| M | 39 | Control-2 |  |
| F | 41 | Control-3 |  |
| M | 46 | Control-4 |  |
| F | 37 | Control-5 |  |
| F | 53 | Control-6 |  |
| F | 51 | Control-7 |  |
| F | 39 | Control-8 | #2 |
| M | 44 | Control-9 |  |
| M | 35 | Control-10 |  |
| F | 28 | Control-11 |  |
| F | 34 | Control-12 |  |
| F | 41 | Control-13 |  |
| M | 23 | Control-14 |  |
| M | 19 | Control-15 | #3 |
| F | 36 | Control-16 |  |
| F | 44 | Control-17 |  |
| M | 51 | Control-18 |  |
| M | 67 | Control-19 |  |
| F | 55 | Control-20 |  |
| F | 33 | Control-21 |  |

Table S3. Sex and age of RAS patients and healthy volunteers included in proteomic analysis.
