# Supplementary material for: Identification of novel serum proteins that distinguish idiopathic recurrent aphthous stomatitis from Behcet’s disease
Source: PeerJ. 2026 Jul 15;14:e21511. doi: 10.7717/peerj.21511 (PMC13380236; doi:10.7717/peerj.21511)
Supplement: Table S4 [file peerj-14-21511-s007.docx]

| Sex | Age | IDs |
| --- | --- | --- |
| F | 45 | RAS-1 |
| F | 38 | RAS-2 |
| M | 55 | RAS-3 |
| F | 39 | RAS-4 |
| M | 53 | RAS-5 |
| M | 44 | RAS-6 |
| M | 29 | RAS-7 |
| F | 48 | RAS-8 |
| F | 62 | RAS-9 |
| M | 65 | RAS-10 |
| F | 62 | RAS-11 |
| F | 56 | RAS-12 |
| F | 51 | RAS-13 |
| F | 32 | RAS-14 |
| M | 44 | RAS-15 |
| M | 72 | RAS-16 |
| F | 39 | RAS-17 |
| M | 67 | RAS-18 |
| F | 41 | RAS-19 |
| F | 47 | RAS-20 |
| M | 43 | RAS-21 |
| F | 32 | RAS-22 |
| M | 29 | RAS-23 |
| M | 60 | RAS-24 |
| F | 56 | RAS-25 |
| F | 42 | RAS-26 |
| F | 33 | Control-1 |
| F | 45 | Control-2 |
| M | 45 | Control-3 |
| F | 46 | Control-4 |
| M | 55 | Control-5 |
| M | 39 | Control-6 |
| F | 62 | Control-7 |
| F | 56 | Control-8 |
| F | 39 | Control-9 |
| F | 41 | Control-10 |
| M | 51 | Control-11 |
| M | 25 | Control-12 |
| M | 33 | Control-13 |
| F | 47 | Control-14 |
| F | 41 | Control-15 |
| F | 32 | Control-16 |
| F | 27 | Control-17 |
| M | 42 | Control-18 |
| F | 53 | Control-19 |
| M | 62 | Control-20 |
| F | 35 | Control-21 |
| F | 44 | Control-22 |
| M | 68 | Control-23 |
| M | 32 | Control-24 |
| M | 41 | Control-25 |
| F | 42 | Control-26 |
| F | 44 | Control-27 |
| M | 34 | Control-28 |
| M | 47 | Control-29 |
| M | 51 | Control-30 |

Table S4. Sex and age of RAS patients and healthy volunteers included in ELISA.
