# Supplementary material for: Identification of novel serum proteins that distinguish idiopathic recurrent aphthous stomatitis from Behcet’s disease
Source: PeerJ. 2026 Jul 15;14:e21511. doi: 10.7717/peerj.21511 (PMC13380236; doi:10.7717/peerj.21511)
Supplement: Table S5 [file peerj-14-21511-s008.docx]

| **Characteristic** | **Control**  **(n = 21)** | **BD**  **(n = 12)** | **RAS**  **(n = 12)** | **Test Statistic** | ***P*-value** |
| --- | --- | --- | --- | --- | --- |
| **Sex, n (%)** |  |  |  | χ² test,  χ² = 0.04 | 0.98 |
| Male | 7 (33.3) | 4 (33.3) | 4 (33.3) |  |  |
| Female | 14 (66.7) | 8 (66.7) | 8 (66.7) |  |  |
| **Age (years)** |  |  |  | One-way ANOVA,  F = 0.47 | 0.63 |
| Mean ± SD | 40.5 ± 11.4 | 44.0 ± 14.4 | 43.8 ± 11.6 |  |  |
| Median [IQR] | 41 [35, 46] | 41 [35, 58] | 48 [33, 51] |  |  |
| Range | 19 - 67 | 23 - 66 | 25 - 65 |  |  |

**Table S5. Comparison of demographic characteristics of the proteomic analysis cohort.**
