# Supplementary material for: Identification of novel serum proteins that distinguish idiopathic recurrent aphthous stomatitis from Behcet’s disease
Source: PeerJ. 2026 Jul 15;14:e21511. doi: 10.7717/peerj.21511 (PMC13380236; doi:10.7717/peerj.21511)
Supplement: Table S6 [file peerj-14-21511-s009.docx]

| **Characteristic** | **Control**  **(n = 30)** | **RAS**  **(n = 26)** | **BD**  **(n = 26)** | **Test Statistic** | ***P*-value** |
| --- | --- | --- | --- | --- | --- |
| **Sex, n (%)** |  |  |  | χ² test,  χ² = 1.32 | 0.52 |
| Male | 13 (43.3) | 10 (38.5) | 9 (34.6) |  |  |
| Female | 17 (56.7) | 16 (61.5) | 17 (65.4) |  |  |
| **Age (years)** |  |  |  | One-way ANOVA,  F = 0.25 | 0.78 |
| Mean ± SD | 43.3 ± 10.7 | 47.2 ± 12.2 | 45.0 ± 13.3 |  |  |
| Median [IQR] | 42.5 [37, 49] | 45.5 [39, 56] | 46 [37, 56] |  |  |
| Range | 25 - 68 | 29 - 72 | 16 - 67 |  |  |

**Table S6. Comparison of demographic characteristics of the ELISA cohort.**
