# Supplementary material for: Identification of novel serum proteins that distinguish idiopathic recurrent aphthous stomatitis from Behcet’s disease
Source: PeerJ. 2026 Jul 15;14:e21511. doi: 10.7717/peerj.21511 (PMC13380236; doi:10.7717/peerj.21511)
Supplement: Table S8 [file peerj-14-21511-s011.docx]

| **Protein** | **Comparison** | **Fold Change** | **Coefficient of variation**  **Healthy**  **(%)** | **Coefficient of variation**  **BD**  **(%)** | **Coefficient of variation**  **RAS**  **(%)** | | **Power (%)** | **α (two-tailed)** |
| --- | --- | --- | --- | --- | --- | --- | --- | --- |
| ENO1 | BD vs Healthy | 1.95× | 14.9 | 8.6 | 10.8 | >99.9 | | 0.05 |
|  | RAS vs Healthy | 1.56× | 14.9 | 8.6 | 10.8 | >99.9 | | 0.05 |
|  | BD vs RAS | 1.26× | 14.9 | 8.6 | 10.8 | >99.9 | | 0.05 |
| S100A7 | BD vs Healthy | 1.70× | 16.5 | 8.1 | 12.2 | >99.9 | | 0.05 |
|  | RAS vs Healthy | 1.34× | 16.5 | 8.1 | 12.2 | >99.9 | | 0.05 |
|  | BD vs RAS | 1.26× | 16.5 | 8.1 | 12.2 | >99.9 | | 0.05 |
| ANXA2 | BD vs Healthy | 2.35× | 19.5 | 7.3 | 11.6 | >99.9 | | 0.05 |
|  | RAS vs Healthy | 1.77× | 19.5 | 7.3 | 11.6 | >99.9 | | 0.05 |
|  | BD vs RAS | 1.32× | 19.5 | 7.3 | 11.6 | >99.9 | | 0.05 |
